# Rapidly cycling Lgr5^+^ stem cells are exquisitely sensitive to extrinsic dietary factors that modulate colon cancer risk

**DOI:** 10.1038/cddis.2016.269

**Published:** 2016-11-10

**Authors:** Eunjoo Kim, Laurie A Davidson, Roger S Zoh, Martha E Hensel, Michael L Salinas, Bhimanagouda S Patil, Guddadarangavvanahally K Jayaprakasha, Evelyn S Callaway, Clinton D Allred, Nancy D Turner, Brad R Weeks, Robert S Chapkin

**Affiliations:** 1 Program in Integrative Nutrition and Complex Diseases, Texas A&M University, College Station, TX, USA; 2 Department of Cellular and Molecular Medicine, Texas A&M Health Science Center, College Station, TX, USA; 3 Center for Translational Environmental Health Research, Texas A&M University, College Station, TX, USA; 4 Department of Epidemiology and Biostatistics, Texas A&M Health Science Center, College Station, TX, USA; 5 Department of Veterinary Pathobiology, Texas A&M University, College Station, TX, USA; 6 Vegetable Crop Improvement Center, Texas A&M University, College Station, TX, USA; 7 Department of Microbial Pathogenesis and Immunology, Texas A&M Health Science Center, College Station, TX, USA

## Abstract

The majority of colon tumors are driven by aberrant Wnt signaling in intestinal stem cells, which mediates an efficient route toward initiating intestinal cancer. Natural lipophilic polyphenols and long-chain polyunsaturated fatty acids (PUFAs) generally suppress Wnt- and NF-*κ*B- (nuclear factor*-κ* light-chain enhancer of activated B-cell) related pathways. However, the effects of these extrinsic agents on colonic leucine-rich repeat-containing G-protein-coupled receptor 5-positive (Lgr5^+^) stem cells, the cells of origin of colon cancer, have not been documented to date. Therefore, we examined the effect of n-3 PUFA and polyphenol (curcumin) combination on Lgr5^+^ stem cells during tumor initiation and progression in the colon compared with an n-6 PUFA-enriched control diet. Lgr5-EGFP-IRES-^creERT2^ knock-in mice were fed diets containing n-6 PUFA (control), n-3 PUFA, n-6 PUFA+curcumin or n-3 PUFA+curcumin for 3 weeks, followed by 6 azoxymethane (AOM) injections, and terminated 17 weeks after the last injection. To further elucidate the effects of the dietary bioactives at the tumor initiation stage, Lgr5^+^ stem cells were also assessed at 12 and 24 h post AOM injection. Only n-3 PUFA+curcumin feeding reduced nuclear *β*-catenin in aberrant crypt foci (by threefold) compared with control at the progression time point. n-3 PUFA+curcumin synergistically increased targeted apoptosis in DNA-damaged Lgr5^+^ stem cells by 4.5-fold compared with control at 12 h and maximally reduced damaged Lgr5^+^ stem cells at 24 h, down to the level observed in saline-treated mice. Finally, RNAseq analysis indicated that p53 signaling in Lgr5^+^ stem cells from mice exposed to AOM was uniquely upregulated only following n-3 PUFA+curcumin cotreatment. These novel findings demonstrate that Lgr5^+^ stem cells are uniquely responsive to external dietary cues following the induction of DNA damage, providing a therapeutic strategy for eliminating damaged Lgr5^+^ stem cells to reduce colon cancer initiation.

Numerous epidemiological studies have established strong evidence that many cancers including colon cancer have substantial risk proportions attributed to extrinsic factors such as diet, smoking and obesity.^1^ High fat diet (HFD)-induced obesity augments stemness and tumorigenicity of intestinal progenitors.^2^ In addition, epithelial colonic leucine-rich repeat-containing G-protein-coupled receptor 5-positive (Lgr5)^+^ stem cells respond to HFD^3^ and genotoxic carcinogen, for example, AOM,^4^ during the cancer initiation stage. It has also been demonstrated that colonic Lgr5^+^ stem cells are preferentially damaged by AOM compared with differentiated cells.^4^ A functional consequence of this status could enhance tumorigenesis. Thus, induction of apoptosis in DNA-damaged Lgr5^+^ stem cells is likely to be a useful marker for successful cancer prevention, and may hold promise for identifying novel and improved cancer-chemopreventive agents. For example, it has been shown that nonsteroidal anti-inflammatory drugs (NSAIDs) can induce apoptosis in Lgr5^+^ stem cells and suppress adenoma formation in APC^Min/+^ mice.^5^ Long-term use of NSAIDs, in particular COX-2-specific inhibitors, is associated with side effects, which has stimulated the development of new targets and combination strategies for cancer chemoprevention.^6^

It is clear that select extrinsic bioactives, for example, n-3 polyunsaturated fatty acids (n-3 PUFAs) and curcumin (Cur), independently reduce aberrant crypt foci (ACF) and polyp formation in humans^7, 8^ and combinations of the two have been reported to exert synergistic effects related to the suppression of chronic inflammation and cell proliferation and the promotion of apoptosis in breast and pancreatic cancers.^9, 10, 11, 12, 13^ From a mechanistic perspective, the combination of n-3 PUFA and curcumin has been shown to suppress NF-*κ*B (nuclear factor*-κ* light-chain enhancer of activated B cell) activation in mouse colonic mucosa,^10^ in part, by altering plasma membrane composition,^14^ which is required for activation of the apoptotic pathways.^15^ The suppression of inflammatory mediators such as COX-2, inducible nitric oxide synthase, prostaglandin E_2_, 5-lipoxygenase and cytosolic phospholipase A_2_ has also been linked to the synergistic action of curcumin and n-3 PUFA, for example, docosahexaenoic acid (DHA).^11, 12^

DHA and curcumin synergistically induce p53 activation,^9, 13^ a well-known tumor suppressor.^16^ This is noteworthy because p53 functions, in part, to inhibit NF-*κ*B activity, thereby inducing apoptosis in human colon cancer cells.^17^ In contrast, the loss of p53 during tumor progression promotes an NF-*κ*B-dependent inflammatory microenvironment,^18^ which is a critical event in the commitment of stem cells to apoptosis following DNA damage.^19, 20^ Unfortunately, the properties of extrinsic chemoprotective natural compounds known to enhance the p53 signaling pathway in colonic adult stem cells from the perspective of cancer initiation and progression have not been determined.

In this study, we assessed the chemoprotective effects of diet on Lgr5^+^ stem cell homeostasis in the context of AOM-induced tumorigenesis at both the initiation and pretumor stages. Our novel data provide evidence that curcumin combined with n-3 PUFA synergistically reduces AOM-induced nuclear *β*-catenin levels in ACF, in part, by promoting p53-dependent signaling and targeted apoptosis in damaged Lgr5^+^ stem cells at the initiation stage. We also provide evidence that DNA-damaged Lgr5^+^ stem cells are highly responsive to the combination diet compared with DNA-damaged differentiated cells. Our findings demonstrate for the first time that rapidly cycling Lgr5^+^ stem cells are exquisitely sensitive to extrinsic dietary factors which modulate colon cancer risk.

## Results

### n-3 PUFA and curcumin synergize to promote targeted apoptosis of damaged Lgr5^+^ stem cells at the tumor initiation stage

To elucidate the unique properties of colonic Lgr5^+^ stem cells in terms of their response to bioactive compounds in the presence of carcinogen, we used mice carrying a gene for enhanced green fluorescent protein (EGFP) knocked into the Lgr5 genomic locus.^24^ Mice were fed with n-3 PUFA and curcumin alone or in combination for 3 weeks, injected with AOM and analyzed to measure DNA damage (gamma H2AX (*γ*H2AX)), apoptosis (terminal deoxynucleotidyl transferase dUTP nick-end labeling (TUNEL)), damage repair (methylguanine-methyl transferase (MGMT)) and proliferation (5-ethynyl-2′-deoxyuridine (EdU)) in crypts 12 and 24 h later as described previously.^4^ Because the integration cassette in this mouse model is epigenetically silent in about 60–70% of crypts,^25^ we analyzed only GFP-positive crypts using fluorescence microscopy. No differences between GFP^high^ and GFP^neg^ crypts in response to carcinogen and bioactive compounds were observed (data not shown). In a separate experiment, levels of n-3 PUFA, n-6 PUFA and curcumin incorporated into colonic crypts were assessed following a 3-week feeding period (Supplementary Figure 1F). The data demonstrate that these bioactives were readily incorporated into the target site.

**Figure 1:**
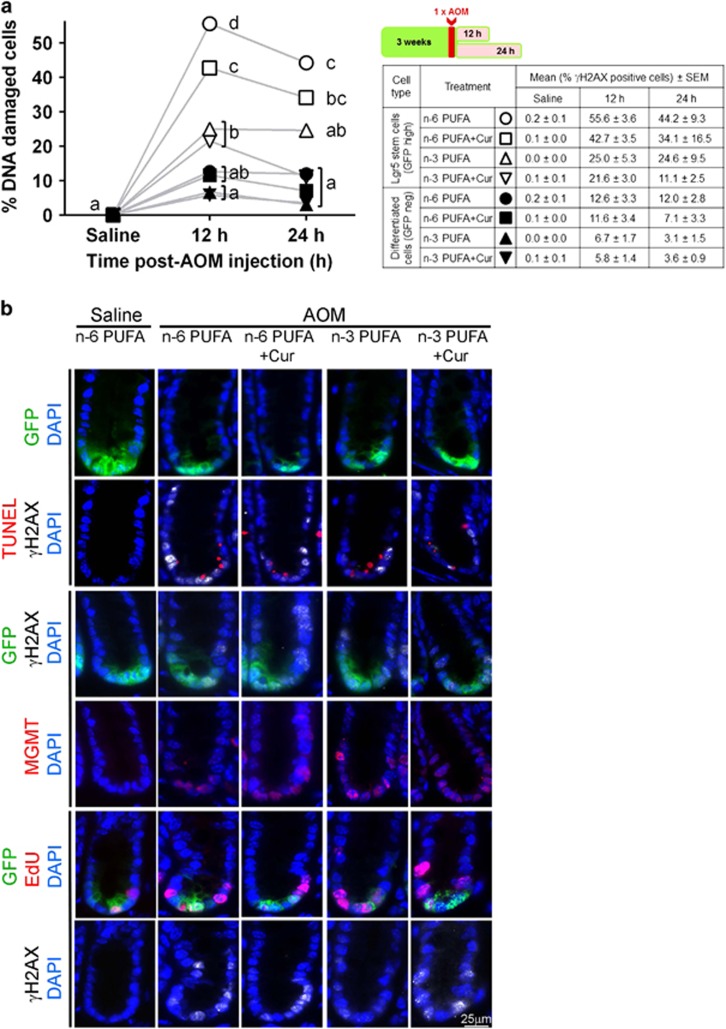
Effect of n-3 PUFA and curcumin on AOM-induced DNA DSBs in mouse colonic crypts at 12 and 24 h post AOM injection. (**a**) Comparison of *γ*H2AX^+^ (DNA-damaged) stem and differentiated cells in the distal colon of saline (control) and 12 and 24 h post AOM-injected mice (left). GFP^+^ crypts from *n*=8 to 9 mice at 12 h, and *n*=3 for saline and 24 h were counted. Statistically significant differences between diets and treatments were determined using two-way analysis of variance (ANOVA), followed by Tukey's multiple comparison test adjustment. Different letters indicate significant differences between treatment groups in each time (*P*<0.05). Means and standard errors of the mean (S.E.M.) in different cell types, diets and treatments are listed in the table (right). Diets are labeled with different symbols and the open symbols refer to stem cells, whereas filled symbols refer to differentiated cells. (**b**) Representative images (objective, × 40) of GFP^+^ (Lgr5 stem cells, green), *γ*H2AX^+^ (DNA DSBs, white) and TUNEL^+^ (apoptotic body, red) cells 12 h after AOM. Representative images of MGMT^+^ (DNA damage repair, red) and EdU^+^ (cell proliferation, red) cells 24 h following AOM exposure. Saline-injected animals serve as the control. n-6, n-6 PUFA; n-6+Cur, n-6 PUFA+curcumin; n-3, n-3 PUFA; n-3+Cur, n-3 PUFA+curcumin

It has been demonstrated that Lgr5^+^ stem cells are preferentially damaged by AOM in the distal colon compared with differentiated cells^4^ and n-3 PUFA reduce AOM-induced DNA adducts,^26^ which cause double-strand breaks (DSBs).^4^ Therefore, to determine the chemoprotective effects of combinatorial bioactives on AOM-induced DNA damage in Lgr5^+^ stem cells as compared with differentiated cells *in vivo*, we measured *γ*H2AX, a marker of the double-strand breaks. As shown in Figure 1a, the level of DNA-damaged stem cells at 12 h in both n-3 PUFA and n-3 PUFA+curcumin treatments was 50% lower compared with the n-6 PUFA. In comparison, DNA damage in n-6 PUFA+curcumin was 33% lower compared with n-6 PUFA-fed mice. Strikingly, by 24 h, the n-3 PUFA+curcumin group maximally decreased damaged Lgr5^+^ stem cells, that is, the level was not different from the saline-injected mice.

**Figure 2:**
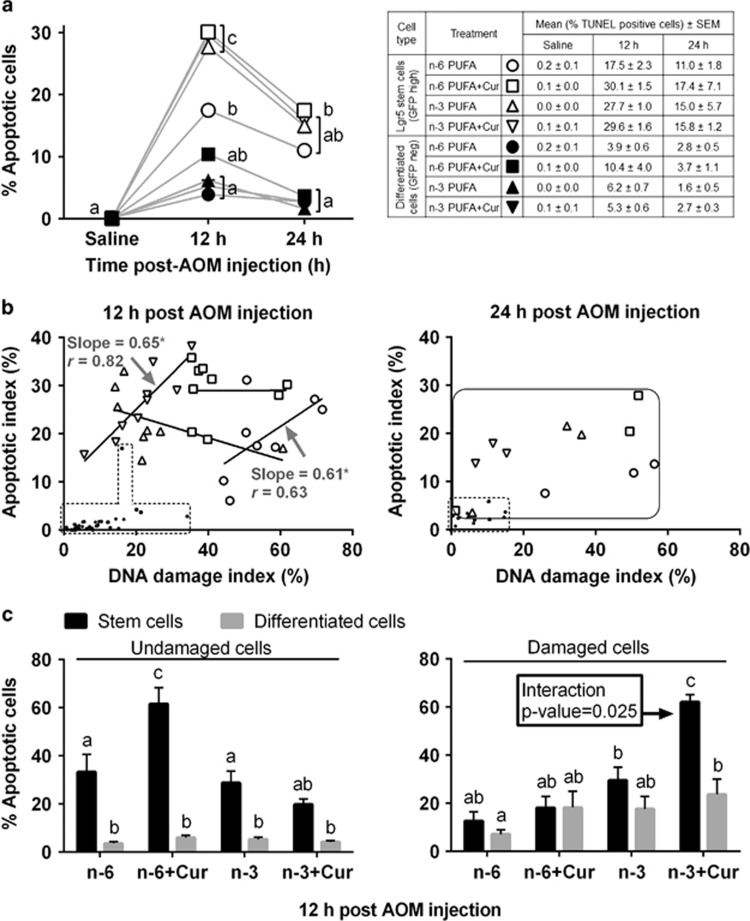
Effect of n-3 PUFA and curcumin on AOM-induced apoptosis in mouse colonic crypts at 12 and 24 h post AOM injection. (**a**) Comparison of TUNEL^+^ (apoptotic) stem and differentiated cells in the distal colon of saline, and 12 and 24 h post AOM-injected mice (left). Refer to Figure 1a legend for animal number and statistical details. Different letters indicate significant (*P*<0.05) differences between treatment groups at each time point. (**b**) Association between AOM-induced apoptotic cells and *γ*H2AX^+^ cells in stem and differentiated cells at 12 h (left) and 24 h (right). Each point represents an individual animal and diets are labeled with different symbols as indicated in the Table in A. Slopes shown are found to be significantly different from 0 at a significant level of 0.05 (n-3 PUFA+Cur and n-6 PUFA stem cells, 12 h). Data from differentiated cells (12 and 24 h) and stem cells (24 h) are highlighted within the dotted lines and solid lines, respectively, instead of showing slope, as none of the slopes differed from zero. Linear regression was performed using GraphPad Prism 6.0. Values represent Pearson's correlation coefficients, *r*, ranges from −1 to +1. *P*-values were calculated using an F-test. Apoptotic index=no. of TUNEL^+^ stem or differentiated cells/total no. of stem or differentiated cells per crypt x100 at 12 and 24 h post AOM injection; damage index=no. of *γ*H2AX^+^ stem or differentiated cells/total no. of stem or differentiated cells per crypt x100 at 12 and 24 h post AOM injection. (**c**) Percentage of non-targeted apoptosis (no. of TUNEL^+^ and *γ*H2AX^−^ stem or differentiated cells/total no. of *γ*H2AX^−^ stem or differentiated cells x100) at 12 h post AOM injection (left). Percentage of targeted apoptosis (no. of double-positive TUNEL^+^ and *γ*H2AX^+^ stem or differentiated cells/total no. of *γ*H2AX^+^ stem or differentiated cells x100) at 12 h post AOM injection (right). Refer to Figure 1a legend for animal numbers and statistics. Bars that do not share the same letter are significantly different (*P*<0.05). n-6, n-6 PUFA; n-6+Cur, n-6 PUFA+curcumin; n-3, n-3 PUFA; n-3+Cur, n-3 PUFA+curcumin

The deletion of damaged Lgr5^+^ stem cells, the cells of origin of colon cancer,^21^ is a critical mechanism to prevent tumorigenesis in the intestine.^5^ As n-3 PUFA^27^ and curcumin^28^ promote apoptosis in 1,2-dimethylhydrazine or AOM-injected rats, we next investigated the synergistic effects of n-3 PUFA+curcumin on the Lgr5^+^ stem cell acute DNA damage response. Figure 1b shows representative colocalization images of DNA-damaged (*γ*H2AX, white) Lgr5^+^ stem cells (GFP, green) with apoptosis (TUNEL, red), DNA repair (MGMT, red) or cell proliferation (EdU, red). As shown in Figure 2a, immediately following AOM exposure, n-3 PUFA and curcumin either alone or in combination increased apoptosis >1.5-fold in Lgr5^+^ stem cells (open symbols) at 12 h compared with n-6 PUFA, whereas the apoptotic response was significantly lower in differentiated cells across all groups (filled symbols). Next, using a simple linear regression analysis, we determined whether there is any association between AOM-induced apoptosis and AOM-induced damage. As shown in Figure 2b (left), n-3 PUFA+curcumin (slope=0.65) and n-6 PUFA (slope=0.61) exhibited a significant proportional increase in apoptosis in Lgr5^+^ stem cells in response to DNA damage at 12 h. However, n-3 PUFA+curcumin exhibited <40% of damaged (*γ*H2AX^+^) Lgr5^+^ stem cells per crypt (DNA damage index), whereas n-6 PUFA had 40–80% of damaged Lgr5^+^ stem cells per crypt. These data suggest that Lgr5^+^ stem cells responded efficiently to n-3 PUFA+curcumin treatment in the presence of DNA damage by inducing apoptosis, whereas differentiated cells were not responsive (small symbols within the dotted line). We subsequently quantified the number of damaged Lgr5^+^ stem cells that were targeted for apoptotic deletion, that is, GFP^+^, TUNEL^+^, *γ*H2AX^+^ triple-positive cells, as the selective deletion of damaged Lgr5^+^ stem cells through apoptosis could mitigate the clonal expansion of damaged Lgr5^+^ stem cells. As shown in Figure 2c (right), 62% of DNA-damaged Lgr5^+^ stem cells were deleted by apoptosis in n-3 PUFA+curcumin-treated mice at 12 h, which was fivefold higher than the n-6 PUFA group. Importantly, n-3 PUFA and curcumin interaction was only detected in Lgr5^+^ stem cells (*P*=0.025) and not in differentiated cells (*P*=0.526) (Supplementary Tables 1A and B). These novel findings demonstrate that DNA-damaged Lgr5^+^ stem cells uniquely respond to n-3 PUFA and curcumin, and n-3 PUFA and curcumin synergize to promote the deletion of DNA-damaged Lgr5^+^ stem cell by enhancing targeted apoptosis.

### AOM-induced MGMT expression is enhanced by n-3 PUFA and/or curcumin in damaged Lgr5^+^ stem cells and differentiated cells

**Figure 3:**
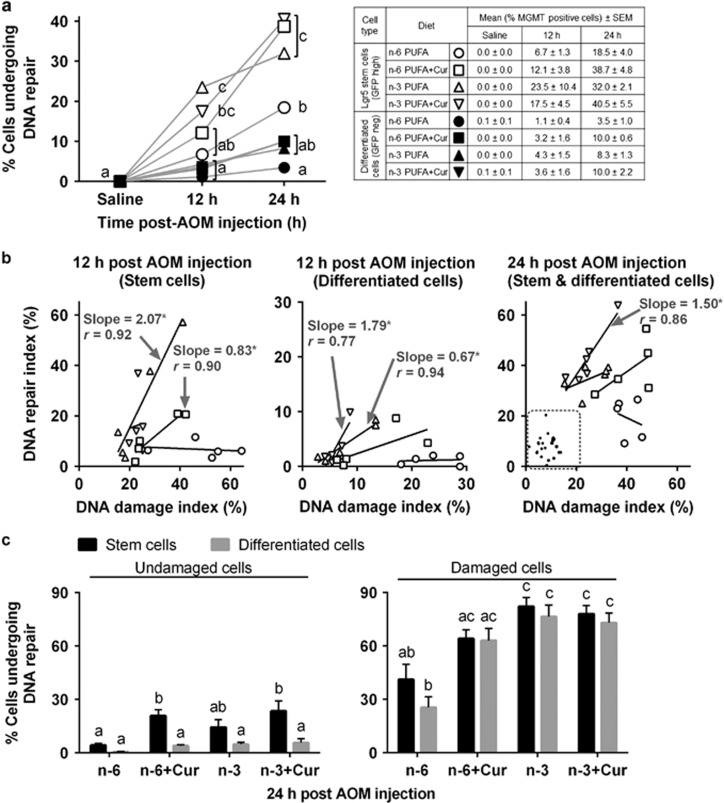
Effect of n-3 PUFA and curcumin on AOM-induced MGMT expression in mouse colonic crypts at 12 and 24 h post AOM injection. (**a**) Comparison of MGMT^+^ (DNA damage repairing) stem and differentiated cells in the distal colon of saline and 12 and 24 h post AOM-injected mice (left). GFP^+^ crypts were scored from *n*=3 per diet in saline-treated mice; *n*=5–6 per diet in AOM-injected mice at 12 and 24 h. Refer to Figure 1a legend for statistical details. Different letters indicate significant (*P*<0.05) differences between treatment groups at each time point. (**b**) Association between AOM-induced MGMT-expressing cells and *γ*H2AX^+^ cells in stem and differentiated cells at 12 h (left and middle) and 24 h (right). Each point represents an individual animal and diets are labeled with different symbols as indicated in the table in (**a**). Slopes shown are found to be significantly different from 0 at a significant level of 0.05. Data from differentiated cells (24 h) are highlighted within the dotted line instead of showing slope because none of the slopes differed from zero. DNA repair index=no. of MGMT^+^ stem or differentiated cells/total no. of stem or differentiated cells per crypt x100 at 12 and 24 h post AOM injection; damage index=no. of *γ*H2AX^+^ stem or differentiated cells/total no. of stem or differentiated cells per crypt x100 at 12 and 24 h post AOM injection. Refer to Figure 2b legend for statistical details. (**c**) Percentage of undamaged cells expressing MGMT (no. of MGMT^+^ and *γ*H2AX^−^ stem or differentiated cells/total no. of *γ*H2AX^−^ stem or differentiated cells x100) at 24 h post AOM injection (left). Percentage of cells expressing MGMT in damaged cells (no. of double-positive MGMT^+^ and *γ*H2AX^+^ stem or differentiated cells/total no. of *γ*H2AX^+^ stem or differentiated cells x100) at 24 h post AOM injection (right). Refer to Figure 1a legend for statistical details. Bars that do not share the same letter are significantly different (*P*<0.05). n-6, n-6 PUFA; n-6+Cur, n-6 PUFA+curcumin; n-3, n-3 PUFA; n-3+Cur, n-3 PUFA+curcumin

The major pathway to remove AOM-induced O^6^-methylguanine (O^6^meG) DNA adducts is via MGMT. Therefore, the expedited elimination of O^6^meG by increasing MGMT activity is likely to be a successful chemoprevention strategy. Because curcumin induces MGMT protein expression in colon cancer cells, we estimated MGMT activity using quantitative immunohistochemical analysis.^4^ As shown in Figure 3a, MGMT expression in Lgr5^+^ stem cells was maximally induced by curcumin with either PUFA diet or the n-3 PUFA treatment at 24 h as compared with saline treatment. However, only n-3 PUFA+curcumin-fed animals exhibited an induction in MGMT associated with DNA damage in Lgr5^+^ stem cells at 24 h using a simple linear regression analysis (Figure 3b, right). In contrast, in differentiated cells (filled symbol), no significant induction was detected at 24 h (Figure 3a). As shown in Figure 3b (left), at 12 h post AOM exposure, n-3 PUFA and n-6 PUFA+curcumin proportionally increased MGMT expression in Lgr5^+^ stem cells (n-3 PUFA: slope=2.07, *r*=0.92 and n-6 PUFA+curcumin: slope=0.83, *r*=0.90), compared with n-3 PUFA and n-3 PUFA+curcumin treatment induction of MGMT in differentiated cells (GFP^neg^) (Figure 3b, middle). We subsequently quantified the number of damaged Lgr5^+^ stem cells that were targeted for damage repair, that is, GFP^+^, MGMT^+^, *γ*H2AX^+^ triple-positive cells, as the deletion of O^6^meG in Lgr5^+^ stem cells by MGMT could mitigate the clonal expansion of DNA-damaged Lgr5^+^ stem cells. As shown in Supplementary Figure 3B (12 h) and Figure 3c (right, 24 h), n-3 PUFA+curcumin and n-3 PUFA induced MGMT expression in damaged Lgr5^+^ stem cells as compared with n-6 PUFA. Additionally, damaged differentiated cells also induced MGMT expression, similar to damaged Lgr5^+^ stem cells both at 12 and 24 h (Supplementary Figures 3B and C).

### Crypt regeneration following AOM exposure

As shown in Figure 1b, proliferating cells were predominantly located at the bottom of crypt where Lgr5^+^ stem cells and transit amplifying (TA) cells reside, together referred to as the stem cell clonogen compartment.^29^ To assess dietary effects on stem cell proliferation, a requirement for crypt regeneration following insult, EdU^+^ cells in the colonic crypt were quantified following AOM-induced DNA damage. The percentage of proliferating cells at 12 h post AOM *versus* saline injection was not affected across all dietary treatments (Supplementary Figure 2A). An increase in cell division at 24 h was only associated with Lgr5^+^ stem cells, that is, not in differentiated TA cells (Supplementary Figure 2A). These findings indicate that colonic Lgr5^+^ stem cells uniquely respond to cues associated with tissue homeostasis. There was no significant association between the proliferative index and the level of DNA damage (Supplementary Figure 2B) and no diet effects were observed with regard to cell proliferation in damaged Lgr5^+^ stem cells at 12 h (Supplementary Figure 3C) and 24 h (Supplementary Figure 2C). Typically, DNA-damaged stem cells in the gut undergo cell cycle arrest and/or apoptosis via p53-mediated signaling.^30, 31^ Therefore, our inability to detect a decrease in cell cycle activity may have been because of the fact that in the C57BL/6 mouse model, proliferation kinetics rebound by ~12 h following intestinal carcinogen exposure.^30^

### Lgr5^+^ stem cell markers are enhanced by carcinogen exposure

To further elucidate the effects of n-3 PUFA+curcumin in the presence of AOM on Lgr5^+^ stem cells, global transcriptional differences in early response genes between sorted GFP^high^ (Lgr5^+^) and GFP^neg^ (differentiated) cells were assessed by RNA sequencing. Mice were fed with the combination of n-3 PUFA and curcumin or control diet (n-6 PUFA) for 3 weeks, injected with AOM or saline and killed 12 h later. Table 1 demonstrates that GFP^high^ cells expressed high levels of Lgr5 and other stem cell markers, for example, Ascl2 and CD44, whereas GFP^neg^ cells expressed high levels of progenitor cell markers, for example, Reg4 and Muc2, as well as Krt20 and Slc26a3 (Table 1). Surprisingly, mRNA levels of crypt base columnar (CBC) cell marker genes^39^ were rapidly altered by extrinsic factors (Table 2). For example, CD44 mRNA levels in GFP^high^ cells were increased by 5.41-fold (in n-6 PUFA) and 2.88-fold (in n-3 PUFA+curcumin) upon AOM exposure, and the enhancement was significantly higher (1.87-fold) in n-6 PUFA *versus* n-3 PUFA+curcumin-fed mice. Msi1 and Agr3 expression was undetectable in GFP^high^ cells isolated from control mice fed n-6 PUFA and treated with saline. In contrast, AOM exposure resulted in the upregulation of Msi1 and Agr3 by 94.92- and 108.51-fold, respectively.

Differentiated cell markers were also modulated by extrinsic factors. For example, Agr2, mainly expressed in progenitor cells, was increased by AOM (1.62-fold) only in GFP^high^ cells in n-6 PUFA-fed mice (FDR<0.05). This finding is relevant because elevated blood mRNA levels of Agr2 and Lgr5 are associated with poor outcome in patients with colorectal cancer.^40^ It is also noteworthy that in GFP^high^ cells from mice fed n-3 PUFA+curcumin, the expression of Prom1/CD133, a colon cancer stem cell marker,^41^ and Cdx2, a prognostic biomarker in stage II and stage III colon cancer,^42^ were not modulated by AOM (Table 2).

### Dietary fish oil and curcumin synergistically enhance p53 signaling in stem cells following AOM exposure

**Figure 4:**
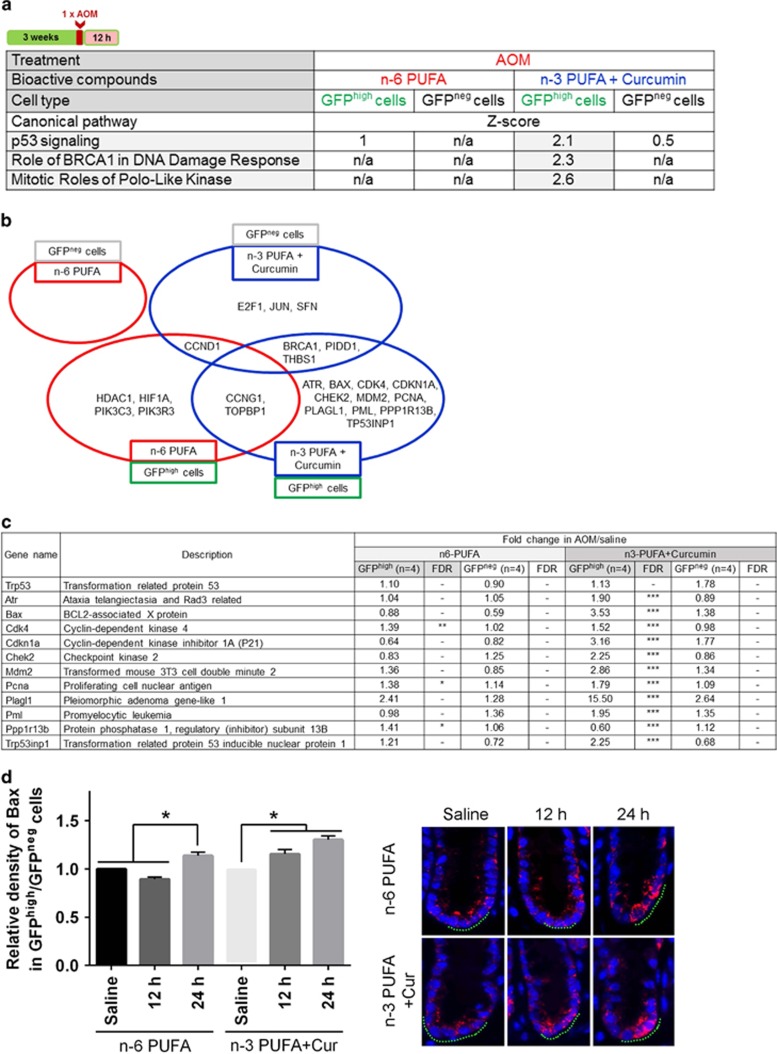
Lgr5^+^ stem cells exclusively enhance p53 signaling pathway by n-3 PUFA+Cur in the presence of AOM. (**a**) Canonical pathways in cells induced by n-6 PUFA or n-3 PUFA+Cur in the presence of AOM. (**b**) P53-associated genes induced by different groups. (**c**) Genes downstream of p53 in GFP^high^ cells uniquely increased by n-3 PUFA+Cur in the presence of AOM. FDR values <0.05 are designated by three asterisks (***), FDR values < 0.1 are designated by two asterisks (**), FDR values <0.2 are designated by one asterisk (*) and any FDR values >0.2 are marked with a dash (−). (**d**, left) Total Bax abundance in cells in n-6 PUFA and n-3 PUFA+Cur. Values were normalized to each respective saline (control) group. *Significant difference between treatment groups (*P*<0.05). (Right) Representative image of Bax-positive (red) cells at 12 h and 24 h post AOM exposure. Green dots indicate the location of GFP-positive Lgr5 stem cells. Saline-injected animals serve as the control. n-6, n-6 PUFA; n-6+Cur, n-6 PUFA+curcumin; n-3, n-3 PUFA; n-3+Cur, n-3 PUFA+curcumin

RNAseq was used to identify signaling pathways that were most significantly modulated by extrinsic cues. From a global transcriptome perspective, GFP^high^ stem cells from n-3 PUFA+curcumin *versus* n-6 PUFA- (control) fed mice treated with AOM exclusively increased p53, BRCA1 and Polo-like kinase-related pathways (*z*-score>1.96) (Figure 4a). This is relevant because rapid accumulation of p53 is associated with cell death in human colon cancer cells.^43^ A total of 24 p53 target genes were increased by extrinsic factors in GFP^high^ and GFP^neg^ cells (Figure 4b). Of the 11 genes uniquely increased by n-3 PUFA+curcumin in GFP^high^ cells, Bax was increased 3.53-fold (Figure 4c). This is noteworthy because human embryonic stem cells (hESCs) have constitutively active Bax at the Golgi, and active Bax translocates from Golgi to mitochondria after DNA damage in a p53-dependent manner, triggering a rapid apoptotic response.^19^ To examine whether the increase in mRNA expression was accompanied by enhanced expression at the protein level, we quantified total Bax expression in Lgr5^+^ stem cells and differentiated cells in n-6 PUFA *versus* n-3 PUFA+curcumin-fed mice in the presence of AOM. As shown in Figure 4d, the relative expression of total Bax in GFP^high^/GFP^neg^ cells was increased 1.3-fold by the administration of n-3 PUFA+curcumin at 12 h post AOM exposure and persisted for up to 24 h (1.5-fold). In contrast, in n-6 PUFA-fed mice, total Bax in GFP^high^/GFP^neg^ cells was unresponsive at 12 h, and increased 1.3-fold at 24 h post AOM injection.

### n-3 PUFA and curcumin combination reduces nuclear *β*-catenin levels in ACF

**Figure 5:**
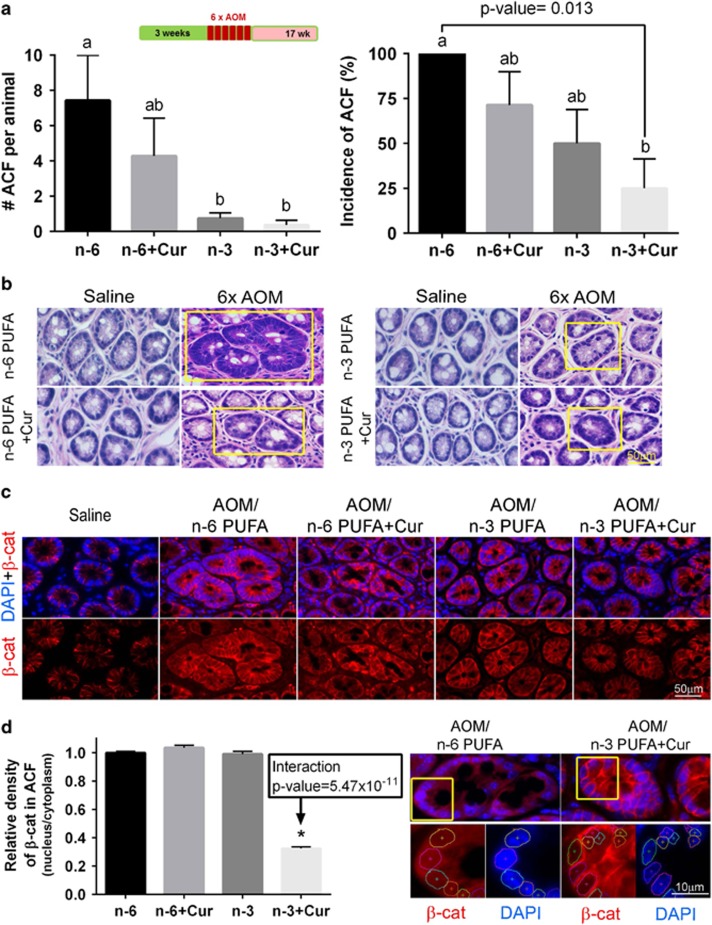
Chemoprotective effects of n-3 PUFA and curcumin on ACF incidence and the subcellular localization of *β*-catenin in ACF. (**a**, left) Percentage of mice with colonic ACF as affected by diet and (right) the number of ACF per animal (half colon) in the presence of AOM. ACF from *n*=7–8 mice were counted 17 weeks after six AOM injections. Bars that do not share the same letter are significantly different (*P*<0.05). (**b**) H&E-stained tissues showing distinct colonic ACF in the center of the image with normal crypts at the periphery in the presence of AOM compared with saline. (**c**) Immunofluorescent images of *β*-catenin (red) in serially sectioned ACF tissue. Nuclear staining was detected by counterstaining cells with DAPI (blue). (**d**, left) Quantification of *β*-catenin expression (staining density) in nucleus/cytoplasm in ACF from mice on the four different diets. Values were normalized to the n-6 PUFA (control) group. (Right) Representative image of *β*-catenin (red) translocated into the nucleus in a n-6 PUFA *versus* n-3 PUFA+Cur-fed mouse. Magnified image of *β*-catenin in the nucleus (nuclear DAPI staining is circled). Refer to Figure 1a legend for statistical details. *Significant difference between treatment groups (*P*<0.05). n-6, n-6 PUFA; n-6+Cur, n-6 PUFA+curcumin; n-3, n-3 PUFA; n-3+Cur, n-3 PUFA+curcumin

Intestinal ACF formation is a surrogate marker of colon cancer both in rodent preclinical models and patients.^44^ Nuclear-to-cytoplasmic *β*-catenin levels are associated with higher mortality in selected groups of colorectal cancer patients^45^ and *β*-catenin accumulated crypts serve as a premalignant biomarker.^46^ As shown in Figure 5a (left), n-3 PUFA±curcumin groups exhibited a significant reduction in the number of ACF per mouse compared to n-6 PUFA and n-3 PUFA+curcumin-treated mice exhibited a significant reduction (75%) in the incidence of ACF as compared with n-6 PUFA (100% ACF incidence). Figure 5b shows representative images of cross-sectioned ACF (yellow boxes) and Figure 5c shows *β*-catenin expression (red) in serial cross-sections of the same ACF. Subsequently, nuclear-to-cytosolic *β*-catenin expression was measured as a biomarker of cancer risk (Figure 5d). *β*-Catenin was predominantly localized to the plasma membrane region of normal colonocytes and negligible nuclear *β*-catenin was detectable in colonic crypt cells in saline-injected animals. In contrast, *β*-catenin immunoreactivity was present in both the cytoplasm and the nucleus in ACF-localized cells as expected^46^ (Figure 5c). Surprisingly, only the n-3 PUFA+curcumin combination treatment exhibited an inhibitory effect (Figure 5d), that is, reduced relative density of nuclear/cytosolic *β*-catenin expression in ACF (n-3 PUFA × curcumin interaction, *P*<0.05) (Supplementary Table 1C). These data indicate that n-3 PUFA+curcumin synergistically suppressed Wnt signaling in ACF, which may underlie the molecular mechanism of n-3 PUFA and curcumin action in the AOM-induced colon cancer model. Unfortunately, the percentage of GFP^+^ Lgr5 cells within the ACF could not be determined due to the mosaic nature of expression in the Lgr5-EGFP-IRES-^creERT2^ knock-in mouse,^25^ that is, a large fraction of ACF were GFP negative.

## Discussion

There is an impending chronic disease crisis in our country and it is predicted that if the current trends continue, the number of cancer cases diagnosed annually by 2050 is likely to double as a result of population aging.^47^ Heading off this escalating burden of age-related illnesses requires an emphasis on primary cancer prevention research and training in cancer-related lifestyle decisions, including diet and exercise.^47^ Here we describe the effect of beneficial dietary factors on DNA damage-induced responses in Lgr5^+^ stem cells in the colon. Further justifying our investigation is the fact that these bioactives do not exhibit the side effects such as gastrointestinal bleeding and myocardial complications associated with the administration of anticancer drugs.^6, 48^

DNA damage induced by AOM is directly linked to colon tumorigenesis in the rodent model. To reduce the risk of tumorigenic transformation of cells, it is critical that DNA-damaged cells be deleted or repaired via MGMT. We have previously demonstrated that colonic Lgr5^+^ stem cells are highly impacted by AOM exposure, resulting in the promotion of apoptosis and induction of MGMT expression compared with differentiated cells during the first 12–24 h after AOM exposure.^4^ In this study, we extend these findings by demonstrating for the first time that n-3 PUFA+curcumin combination maximally promotes targeted apoptosis (Figure 2c) and the induction of MGMT expression in DNA-damaged Lgr5^+^ stem cells (Figure 3b). Consistent with this observation, it has been reported that n-3 PUFA-enriched diets enhance targeted apoptosis during colon tumor initiation, in part, by downregulating Bcl-2.^27^ In addition, n-3 PUFA may prime colonocytes for damage-induced apoptosis by enhancing the unsaturation of mitochondrial phospholipids, resulting in an increase in oxidative stress and the initiation of the apoptotic cascade.^49, 50^ Curcumin can also induce apoptosis by activating mitochondria-initiated apoptotic pathways.^51^ Interestingly, the combination of DHA and curcumin has an additive effect with regard to the induction of apoptosis in breast cancer and pancreatic cell lines.^9, 12^ With respect to Lgr5^+^ stem cell proliferation, only a mild increase was observed at 24 h post AOM as compared with saline. This finding is consistent with the fact that the crypt must regenerate following the loss of cells in the crypt bottom after carcinogen and radiation exposure.^29^

Only mice treated with the combination of n-3 PUFA+curcumin exhibited an induction of p53 signaling exclusively in Lgr5^+^ stem cells in the presence of AOM. This was exemplified, in part, by the induction of Bax at the mRNA and protein levels, a major mediator of p53-mediated apoptosis. These findings are relevant in view of the fact that hESCs have constitutively active Bax at the Golgi and are primed to undergo rapid apoptosis in a p53-dependent manner.^19^ It has also been demonstrated that the absence of an acute apoptotic response to AOM in p53-deficient mice is associated with an increased tumor incidence.^20, 52^ Further studies are needed to elucidate the combinatorial effects of diet on the translocation of active Bax from Golgi to mitochondria. Consistent with these observations, curcumin combined with n-3 PUFA synergistically reduced AOM-induced nuclear *β*-catenin levels in ACF at the pretumor stage. This is noteworthy because elevated nuclear *β*-catenin levels are associated with higher mortality in selected groups of colorectal cancer patients,^45, 53^ and various attempts have been made to identify and characterize pharmacological inhibitors of *β*-catenin.^54^ Consistent with this notion, it has been shown that n-3 PUFA inhibits nuclear location of *β*-catenin in colon cancer cells.^55^

The potential benefits of using a combination of pleiotropic chemoprotective compounds may also be linked to their combined ability to suppress NF-*κ*B activation, which drives COX-2 expression.^14^ In terms of disease progression, COX-2 is capable of enhancing cell proliferation, angiogenesis, cell migration and invasion, as well as inhibiting apoptosis and enhancing tumor growth via cross-talk between Frizzled and the epidermal growth factor receptor (EGFR).^56^ In addition, both DHA and curcumin are capable of modulating plasma membrane structure and EGFR function,^57, 58^ which regulates colon cancer stem cell proliferation. Curcumin can also inhibit the expression of EGFR in human colon cancer cells.^57^

In summary, our data broaden and redefine the phenotypic features of the colonic adult stem cell in the presence of external cues. DNA-damaged Lgr5^+^ stem cells uniquely respond to the combination of n-3 PUFA and curcumin, which promote targeted apoptosis, in part, by enhancing p53 signaling and maximally reducing nuclear *β*-catenin in ACF. This is a critical area of research because long-lived Lgr5^+^ stem cells are responsible for maintaining and regenerating intestinal crypts.^29^ These novel findings demonstrate that Lgr5^+^ stem cells are uniquely responsive to external dietary cues following the induction of DNA damage during tumor initiation and progression, providing a therapeutic strategy for eliminating damaged stem cells and reducing colon cancer risk.

## Materials and Methods

### Animals and study design

The animal use protocol was approved by the University Animal Care Committee of Texas A&M University and conformed to NIH guidelines. Lgr5-EGFP-IRES^creERT2^ knock-in mice,^21^ 6–7 weeks old, were acclimated for 1 week and then maintained on a semipurified diet (Supplementary Figure 1A) for 3 weeks before injection with AOM (Sigma, St. Louis, MO, USA; 10 mg/kg body weight). Mice were injected with AOM once a week for 6 weeks and killed by CO_2_ asphyxiation at 17 weeks (*n*=7–8 per group) after the last AOM injection (Supplementary Figure 1B). In complementary experiments, mice were injected with a single dose of AOM and killed 12 (*n*=8) or 24 h (*n*=8) later (Supplementary Figure 1C). Control mice (*n*=3) received a single saline injection. Mice were injected with EdU (Life Technologies, Grand Island, NY, USA) 2 h before killing. Immediately after termination, the distal (3 cm proximal to the anus) colon was rapidly removed, flushed with ice-cold saline and a longitudinal section of the distal colon was immediately fixed in 4% paraformaldehyde for hematoxylin and eosin (H&E) staining and immunofluorescence analyses.

### Diets

A complete diet containing 5% corn oil (containing n-6 PUFA) was used as a baseline control, that is, contained no n-3 PUFA or curcumin. The control diet was supplemented with 1% (w/w) curcumin (n-6 PUFA+curcumin) to determine the effect of curcumin. A diet containing 4% (w/w) Menhaden fish oil (enriched in n-3 PUFA) was used to assess the effect of n-3 PUFA alone, whereas a fish oil+curcumin (n-3 PUFA+curcumin) diet was used to determine the additive/synergistic effects of these dietary bioactives. Both n-3 PUFA and n-3 PUFA+curcumin diets contained 1% corn oil (w/w) to ensure that essential fatty acid requirements were met (Supplementary Figure 1A). Mice were provided with fresh diet every day and the feeders were removed and washed daily. Animals had free access to food and water at all times and 24 h food intakes were measured after 1 week of receiving the diets (Supplementary Figure 1D). Body weights were recorded each week (Supplementary Figure 1E) and weight gain was not affected by the experimental diets. The fatty acid composition of the diets was analyzed by gas chromatography and the level of curcumin was quantified by ^1^H NMR. The fatty acid composition of the crypt was quantified by gas chromatography and the level of curcumin was quantified by HPLC.

### *In vivo* DNA damage and repair measurement

Longitudinal sections of paraffin-embedded colon sections (4 *μ*m) were deparaffinized, rehydrated through graded ethanol and stained with antibodies using standard procedures. DNA double-strand breaks were measured by immunofluorescence using a rabbit monoclonal phospho-*γ*H2AX Ser139 antibody (9718; Cell Signaling, Danver, MA, USA; dilution 1:200). Lgr5^+^ stem cells were labeled using goat polyclonal GFP antibody (ab6673; Abcam, Cambridge, MA, USA; dilution 1:400) and O^6^meG DNA adduct removal was estimated by the induction of O^6^-MGMT expression using a mouse monoclonal MGMT antibody (ab54306, Abcam; prediluted). Secondary antibodies were anti-rabbit Alexa 647 (711-605-152; Jackson ImmunoResearch, West Grove, PA, USA; dilution 1:400) for *γ*H2AX, anti-goat 488 (705-545-147; Jackson ImmunoResearch) for GFP and anti-mouse Alexa 546 (A10036; Life Technologies) for MGMT. Negative control slides were incubated without primary antibody. The DNA damage (or repair) index was determined by dividing the number of *γ*H2AX^+^ (or MGMT^+^) cells by the total number of cells in each crypt column and multiplying by 100.

### Slide scoring of *in vivo* apoptosis

To investigate whether alkylating agent-induced DNA damage resulted in apoptotic cell death in colonic Lgr5^+^ stem cells, free 3′-OH DNA termini were labeled using the TUNEL procedure according to the manufacturer's recommendations using the TACS 2 TdT-Fluor *In Situ* Apoptosis Detection Kit (4810-30-K and 4810-30-R; Trevigen, Gaithersberg, MD, USA) and detected with Streptavidin-CY3 (438315; Life Technologies). Negative control slides were incubated without TdT enzyme. The apoptotic index was determined by dividing the number of apoptotic cells by the total number of cells in the crypt column and multiplying by 100. To corroborate the frequency of epithelial cells undergoing apoptosis, paraffin-embedded sections were also assessed by H&E staining by a blinded pathologist with both assays showing similar results.

### *In vivo* measurement of cell proliferation

To investigate the effects of alkylating agent-induced DNA damage on cell cycle activity in colonic epithelial cells, proliferating cells were measured using the Click-iT EdU Alexa Fluor 555 Imaging Kit (Life Technologies) as per the manufacturer's instructions. Negative control slides were incubated without Alexa Fluor. The proliferation index was determined by dividing the number of proliferating cells by the total number of cells in the crypt column and multiplying by 100.

### *In vivo* measurement of Bax expression

To measure the chemoprotective effect of diet and carcinogen on Bax expression in the colon, Lgr5^+^ stem cells were labeled using goat polyclonal GFP antibody (ab6673; Abcam; dilution 1:400) and rabbit monoclonal total Bax antibody (14796; Cell Signaling; dilution 1:400). Secondary antibodies were anti-goat 488 (705-545-147; Jackson ImmunoResearch) for GFP and anti-rabbit Alexa 647 (711-605-152; Jackson ImmunoResearch: dilution 1:400) for Bax. Negative control slides were incubated without Alexa Fluor. For each high power field (objective, × 40), GFP-positive crypts were assessed by defining regions of interest for analysis. GFP staining was used to define the region of interest and to assess differentiated cells and stem cells within a crypt. The ratio of Bax expression in Lgr5^+^ stem/differentiated cells was determined using the NIS Image software, version 3.2 (Nikon, Melville, NY, USA).

### Image acquisition and analysis

All immunofluorescent images of colonic crypts were captured using an inverted TE 300 Nikon Eclipse fluorescence microscope equipped with × 40/1.30 Nikon Plan Fluor oil immersion objective and a Photometrics Cool Snap EZ digital CCD camera and a SOLA external light source (Nikon, Melville, NY, USA). Images were processed using the NIS Image software, version 3.2 (Nikon). Greater than 40 GFP-positive crypts per animal in each treatment group were examined by one reader. The number of animals used for each assay is described in each figure legend.

### Cell sorting and RNA sequencing

To examine the global transcriptome in stem *versus* differentiated cells, isolated colonocytes from the distal colon were sorted based on GFP expression using a Beckman Coulter MoFlo (Indianapolis, IN, USA). Approximately 12 000 GFP^high^ cells (stem cells) and 275 000 GFP^neg^ cells (differentiated cells) were sorted for each treated mouse. Consistent with previous observations,^22^ ~98% of costained isolated colonocytes were epithelial cell adhesion molecule (EpCam) positive (data not shown). Isolated total RNA (2 ng) from each sample was subsequently prepared for sequencing using the NuGen Ovation Single Cell RNAseq System (0342HV). Samples were sequenced for 75 cycles with single index reads on the NextSeq 500 (Illumina, San Diego, CA, USA). Following the removal of all genes with CPM (counts per million reads) values <1, publicly available R software, EdgeR, was used to identify differentially expressed genes. In total, 15 043 genes were queried to detect differentially expressed genes. For computational purposes, the EdgeR program adjusts all zero counts to values larger than zero, so log fold changes can be computed.

### Ingenuity pathway analysis

Genes differentially expressed (FDR < 0.05) in GFP^high^ *versus* GFP^neg^ cells were included in pathway and function analyses using the Ingenuity software (ingenuity pathway analysis (IPA); Ingenuity Systems; http://www.ingenuity.com). Statistical significance of the association between each data set and the canonical pathway was determined based on two parameters: (1) a ratio of the number of genes from the data set that map to the pathway divided by the total number of genes that map to the canonical pathway, and (2) *P*-values calculated using Fischer's exact test determining the probability that the association between the genes in the data set and the canonical pathway is due to chance alone. The upstream regulator analysis function of IPA was subsequently used to identify potential transcriptional regulators that could explain the observed changes in gene expression. The activation *z*-score was calculated to predict activation or inhibition of transcriptional regulators based on published findings accessible through the Ingenuity knowledge base. Regulators with a *z*-score >1.96 or <−1.96 were considered to be significantly activated or inhibited. Functions and pathways with *P*-value  <0.05 (Fischer's exact test) were considered to be statistically significant.

### ACF quantification

Paraffin-embedded, H&E-stained distal colon sections (4 *μ*m) were examined for ACF by a veterinary pathologist. Photomicrographs were taken at × 40. ACF were defined as crypts with more than one of the following features: loss of polarity, nuclear atypia (enlarged nuclei, vesiculated chromatin, crowding), mucin depletion and crypt multiplicity. Emphasis was placed on aberrant crypt multiplicity as a cardinal change. The presence of ACF adjacent to lymphoid follicles were not considered true ACF because proximity to lymphoid tissue may promote changes related to inflammation. For this study, only crypts with criteria matching Group C (dysplastic microadenomas, with enlarged, elongated and sometimes stratified nuclei with loss of polarity, mucin depletion and dysplasia)^23^ were considered ACF. The number of ACF per section was quantified and the location mapped using mm measurements from the slide corner. In addition, complementary immunohistochemistry was performed to assess colabeling with *β*-catenin.

### *β*-Catenin subcellular localization in the nucleus and cytoplasm in ACF

Serial sections were used to measure the subcellular localization of *β*-catenin in ACF. To measure the ratio of nuclear-to-cytosolic *β*-catenin in ACF, tissue was labeled using a mouse monoclonal *β*-catenin antibody (610154; BD Transduction, San Joes, CA, USA; dilution 1:500). Secondary antibody was anti-mouse Alexa 546 (A10036; Life Technologies). Nuclear staining was detected by counterstaining cells with 4', 6-diamidino-2-phenylindole (DAPI) (P36935; Life Technologies). Negative control slides were incubated without primary antibody. For each high power field (objective, × 40), ACF areas stained with *β*-catenin and DAPI were assessed by defining regions of interest for analysis. DAPI staining was used to define the nuclear region of interest and to separate nuclear and cytoplasmic staining within a cell. The ratio of nuclear-to-cytosolic *β*-catenin was measured using the NIS Image software, version 3.2 (Nikon).

### Statistics

Data were analyzed using two-way analysis of variance and the estimated mean groups were subsequently compared using simple *t*-tests. A Tukey's approach was used to adjust the resulting *P*-values for multiple comparisons. The significant comparisons were obtained based on the adjusted *P*-values <0.05.

## Figures and Tables

**Table 1:** Differentially expressed marker genes in GFP^high^ *versus* GFP^neg^ colonocytes

*References*	*Cell markers*	*Gene name*	*Description*	*Fold change of GFP*^*high*^*/GFP*^*neg*^ *cells in distal colon*	*FDR*
Munoz *et al.* (2012)	CBC cells	*Ascl2*	*Achaete-scute family bHLH transcription factor 2*	8.08	1.60E−15
		*Lgr5*	*Leucine-rich repeat-containing G-protein-coupled receptor 5*	8.27	1.00E−132
		*Nr2e3*	*Nuclear receptor subfamily 2 group E member 3*	5.58	6.00E−04
		*Tnfrsf19*	*Tumor necrosis factor receptor superfamily, member 19*	2.08	3.70E−01
					
Munoz *et al.* (2012)	Intestinal stem cell signature-CBC cells restricted (mRNAs and proteins)	*Aqp4*	*Aquaporin 4*	0.36	7.70E−46
		*Ascl2*	*Achaete-scute family bHLH transcription factor 2*	8.08	1.60E−15
		*Cd44*	*CD44 antigen*	4.82	3.90E−75
		*Cdca7*	*Cell division cycle-associated 7*	3.4	8.20E−41
		*Cdk6*	*Cyclin-dependent kinase 6*	3.12	1.50E−45
		*Clca4*	*Chloride channel accessory 4*	5.39	2.80E−144
		*Kcnq1*	*Potassium voltage-gated channel, subfamily Q, member 1*	2.84	4.50E−44
		*Msi1*	*Musashi RNA-binding protein 1*	0.77	1.00E+00
		*Nav1*	*Neuron navigator 1*	1.84	2.90E−06
		*Smoc2*	*SPARC-related modular calcium binding 2*	6.24	6.20E−48
		*Soat1*	*Sterol O-acyltransferase 1*	2.06	4.80E−21
	Intestinal stem cell signature-gradient within the crypt with highest expression at the crypt bottom (mRNAs and proteins)	*Afap1l1*	*Actin filament-associated protein 1-like 1*	2.2	1.10E−12
		*Agr3*	*Anterior gradient 3*	1.16	9.30E−01
		*Cnn3*	*Calponin 3, acidic*	3.07	5.20E−30
		*Dach1*	*Dachshund 1*	3.38	4.90E−21
		*Slc12a2*	*Solute carrier family 12, member 2*	2.43	2.20E−71
		*Slco3a1*	*Solute carrier organic anion transporter family, member 3a1*	0.15	2.80E−02
		*Sorbs2*	*Sorbin and SH3 domain containing 2*	2.23	6.60E−30
		*Tns3*	*Tensin 3*	1.74	4.80E−14
		*Vdr*	*Vitamin D receptor*	1.09	1.10E−01
					
Munoz *et al.* (2012)	Quiescent/+4 stem cell markers expressed in CBC cells	*Bmi1*	*Bmi1 polycomb ring-finger oncogene*	1.36	9.10E−03
					
Li *et al.* (2014)		*Hopx*	*HOP homeobox*	2.09	1.20E−13
		*Lrig1*	*Leucine-rich repeats and immunoglobulin-like domains 1*	2.8	1.30E−29
		*Tert*	*Telomerase reverse transcriptase*	1.57	2.80E−01
					
Li *et al.* (2014)	Wnt target genes	*Ascl2*	*Achaete-scute family bHLH transcription factor 2*	8.08	1.60E−15
		*Axin 2*	*Axin 2*	3.72	8.20E−122
		*Sox9*	*SRY (sex determining region Y)-box 9*	1.88	4.90E−39
					
Fevr *et al.* (2007)	TA cells	*Axin 2*	*Axin 2*	3.72	8.20E−122
					
ten Kate *et al.* (1989)		*Ccnd1*	*Cyclin D1*	1.37	1.20E−08
		*Cd44*	*CD44 antigen*	4.82	3.90E−75
		*Myc*	*Myelocytomatosis oncogene*	2.85	8.60E−30
					
Grun *et al.* (2015)	Absorptive enterocytes	*Alpi*	*Alkaline phosphatase, intestinal*	0.06	2.80E−01
	Hormone-secreting enteroendocrine	*Cck*	*Cholecystokinin*	0.9	6.30E−01
		*Chga*	*Chromogranin A*	0.23	3.40E−01
		*Chgb*	*Chromogranin B*	0.47	3.60E−91
		*Reg4*	*Regenerating islet-derived family, member 4*	0.4	6.70E−10
		*Tac1*	*Tachykinin 1*	0.81	9.70E−01
		*Tph1*	*Tryptophan hydroxylase 1*	0.01	3.40E−20
	Tuft cells	*Cd24a*	*CD24a antigen*	1.45	5.20E−20
		*Dclk1*	*Doublecortin-like kinase 1*	1.21	2.30E−46
		*Krt8*	*Keratin 8*	0.4	4.70E−01
		*Krt18*	*Keratin 18*	1.21	6.70E−18
	Goblet cells	*Agr2*	*Anterior gradient 2*	0.89	8.50E−16
		*Clca3*	*Chloride channel accessory 1*	0.13	6.30E−01
		*Dll1*	*Delta-like 1*	0.74	8.30E−59
					
Rothenberg *et al.* (2012)		*Dll4*	*Delta-like 4*	1.32	1.20E−03
Dalerba *et al.* (2012)		*Kit*	*Kit oncogene*	1.09	9.30E−02
		*Muc2*	*Mucin 2*	0.62	3.00E−172
	Genes highly expressed with CD66a	*Ceacam1*	*Carcinoembryonic antigen-related cell adhesion molecule 1*	0.64	1.70E−09
		*Krt20*	*Keratin 20*	0.15	6.17E−61
		*Slc26a3*	*Solute carrier family 26, member 3*	0.15	2.95E−65
	Genes highly expressed with CD44	*Mki67*	*Marker of proliferation Ki-67*	3	9.76E−71
		*Myc*	*Myelocytomatosis oncogene*	2.85	8.62E−30

Abbreviations: CBC, crypt base columnar; FDR, false discovery rate; GFP, green fluorescent protein; TA, transit amplifying.

Fold change of cell type marker gene expression by RNA sequencing of colonic GFP^high^ and GFP^neg^ cells at 12 h post saline exposure, *n*=8 mice per group

**Table 2:**
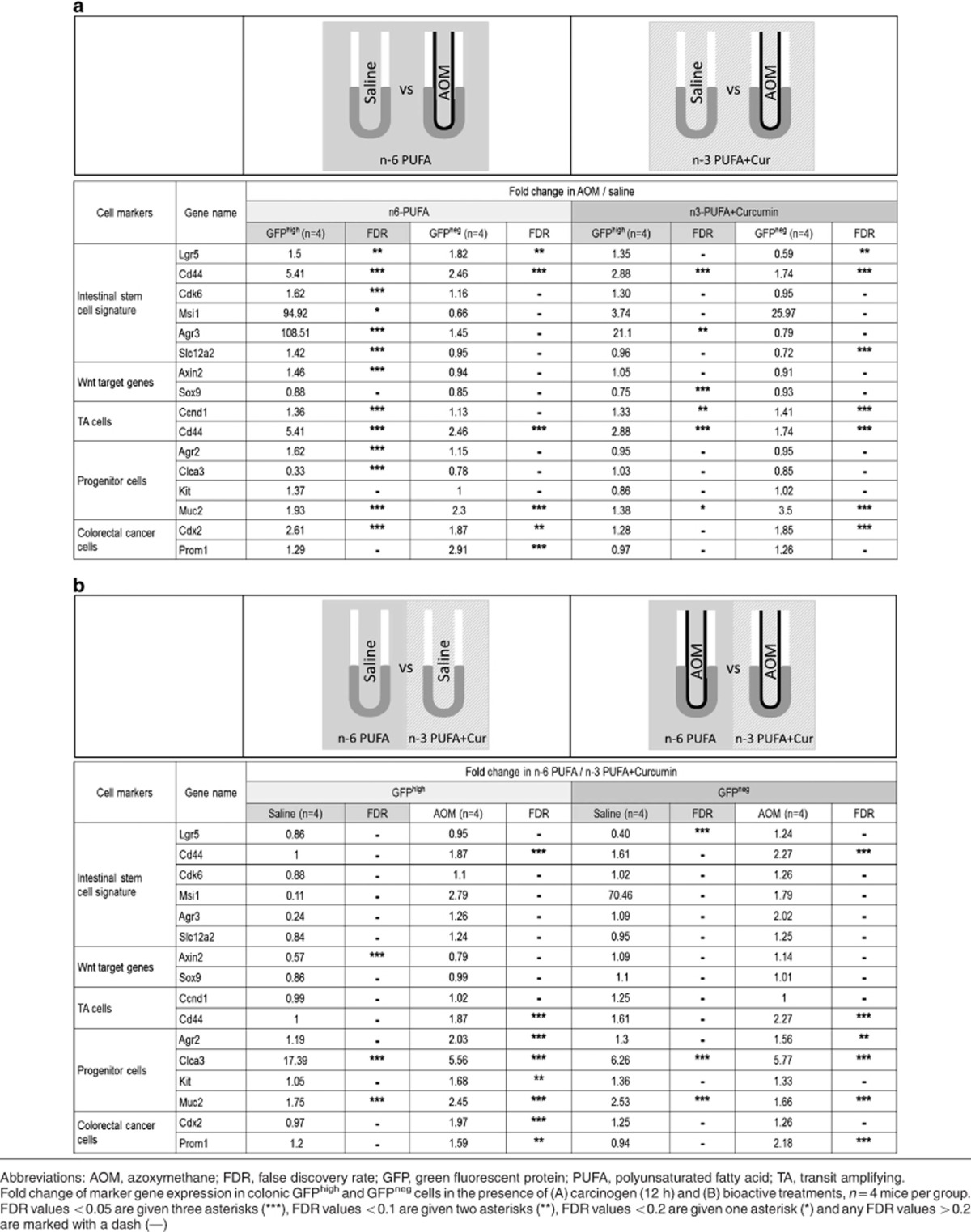
Marker genes transcriptionally modulated by extrinsic factors

## Abbreviations

ACF: aberrant crypt foci
AOM: azoxymethane
DHA: docosahexaenoic acid
EdU: 5-ethynyl-2′-deoxyuridine
EGFP: enhanced green fluorescent protein
*γ*H2AX: gamma H2AX
H&E: hematoxylin and eosin
hESCs: human embryonic stem cells
HFD: high fat diet
IPA: ingenuity pathway analysis
Lgr5: leucine-rich repeat-containing G-protein-coupled receptor 5
MGMT: O6-methylguanine-methyl transferase
NF-*κ*B: nuclear factor*-κ* light-chain enhancer of activated B cells
NSAIDs: nonsteroidal anti-inflammatory drugs
O^6^meG: O6-methylguanine
PUFA: polyunsaturated fatty acids
TUNEL: terminal deoxynucleotidyl transferase dUTP nick-end labeling
