# Supplementary material for: Rapidly cycling Lgr5+ stem cells are exquisitely sensitive to extrinsic dietary factors that modulate colon cancer risk
Source: Cell Death Dis. 2016 Nov 10;7(11):e2460–. doi: 10.1038/cddis.2016.269 (PMC5260883; doi:10.1038/cddis.2016.269)
Supplement: Supplementary Figures and Tables [file cddis2016269x1.pdf]

A

Ingredients	Diet (g/100 g)			
	n-6 PUFA	n-6 PUFA+Cur	n-3 PUFA	n-3 PUFA+Cur
Sucrose	42	41	42	41
Casein	20	20	20	20
DL-methionine	0.3	0.3	0.3	0.3
AIN-76 Mineral mix	3.5	3.5	3.5	3.5
AIN-76 Vitamin mix	1	1	1	1
Choline Chloride	0.2	0.2	0.2	0.2
Corn Starch	22	22	22	22
Cellulose	6	6	6	6
Curcumin	0	1	0	1
Fish oil	0	0	4	4
Corn oil	5	5	1	1
Total	100	100	100	100

B

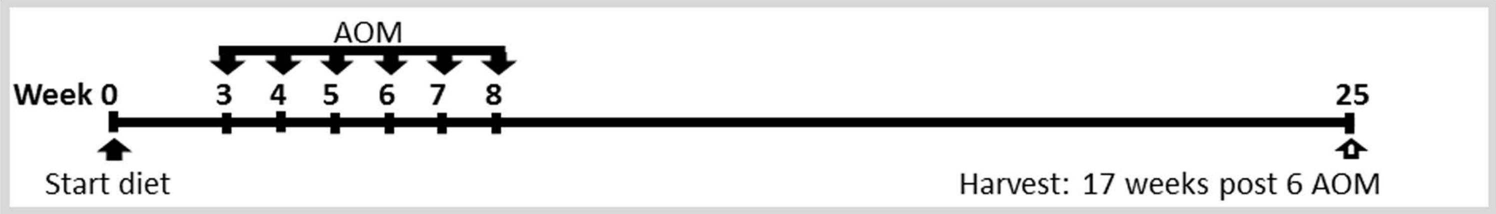

C

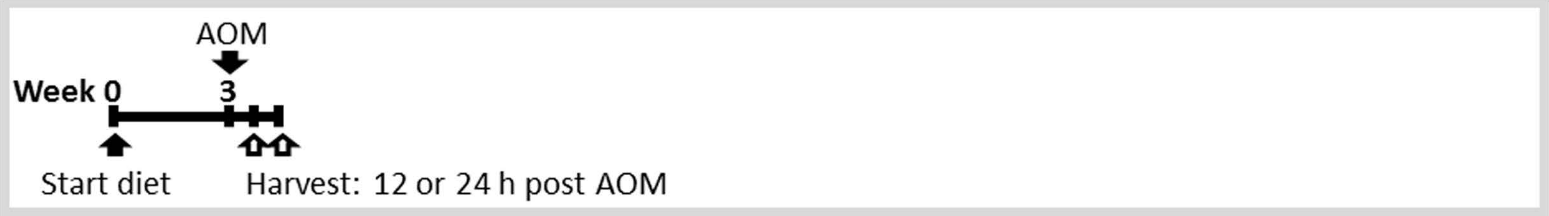

D

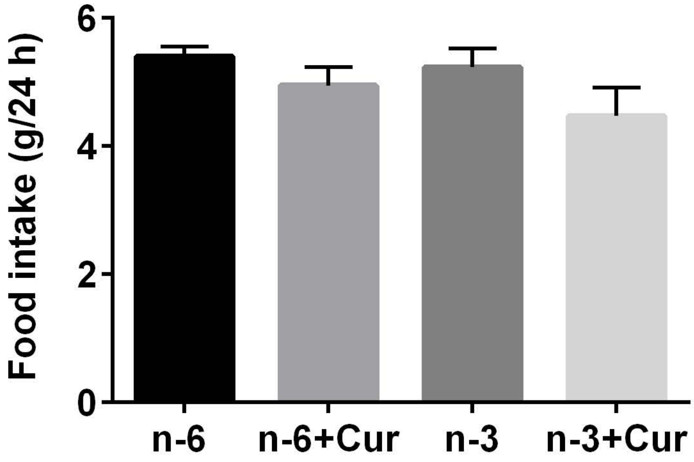

E

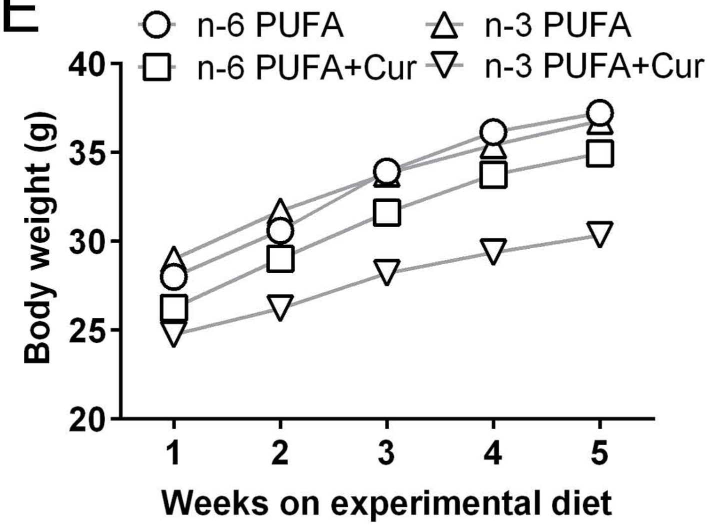

F

Bioactives in mouse experimental diet		n-6 PUFA	n-6 PUFA +Cur	n-3 PUFA	n-3 PUFA +Cur
Fatty acids (ug/g crypt wet weight)	20:5n-3 (EPA)	-	-	19.29 ± 4.54	43.29 ± 11.97
	22:5n-3 (DPA)	-	-	0.80 ± 0.30	1.96 ± 0.56
	22:6n-3 (DHA)	-	-	7.92 ± 1.77	19.34 ± 5.83
Curcumin (ug/g crypt wet weight)		-	4.26 ± 0.95	-	1.08 ± 0.08

Supplemental Figure 1

A

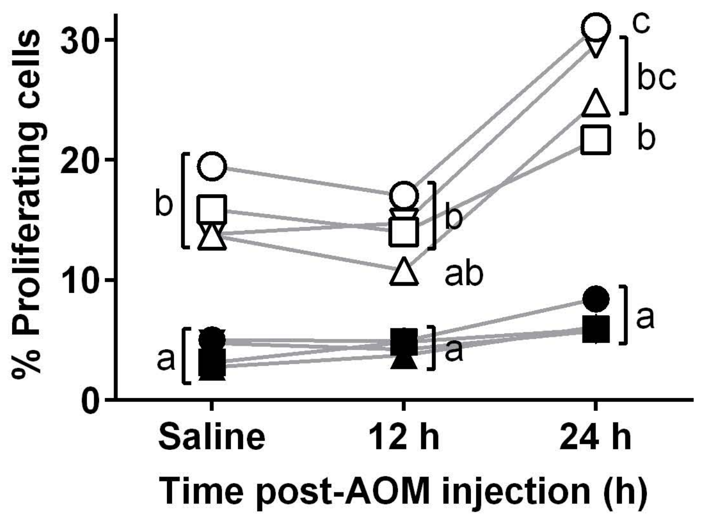

Cell type	Diet		Mean (% EdU positive cells) ± SEM		
			Saline	12 h	24 h
Lgr5 stem cells (GFP high)	n-6 PUFA	○	19.5 ± 1.4	17.0 ± 1.0	31.1 ± 1.6
	n-6 PUFA+Cur	□	15.9 ± 2.1	14.0 ± 1.8	21.7 ± 5.0
	n-3 PUFA	△	13.7 ± 1.1	10.8 ± 1.4	24.8 ± 3.4
	n-3 PUFA+Cur	▽	13.8 ± 1.5	14.8 ± 1.9	29.6 ± 4.1
Differentiated cells (GFP neg)	n-6 PUFA	●	5.0 ± 0.6	4.9 ± 0.8	8.4 ± 0.9
	n-6 PUFA+Cur	■	3.1 ± 0.6	4.8 ± 1.1	5.9 ± 0.6
	n-3 PUFA	▲	2.8 ± 0.4	3.8 ± 1.0	6.1 ± 0.5
	n-3 PUFA+Cur	▼	4.8 ± 0.7	4.2 ± 0.9	5.8 ± 0.6

B

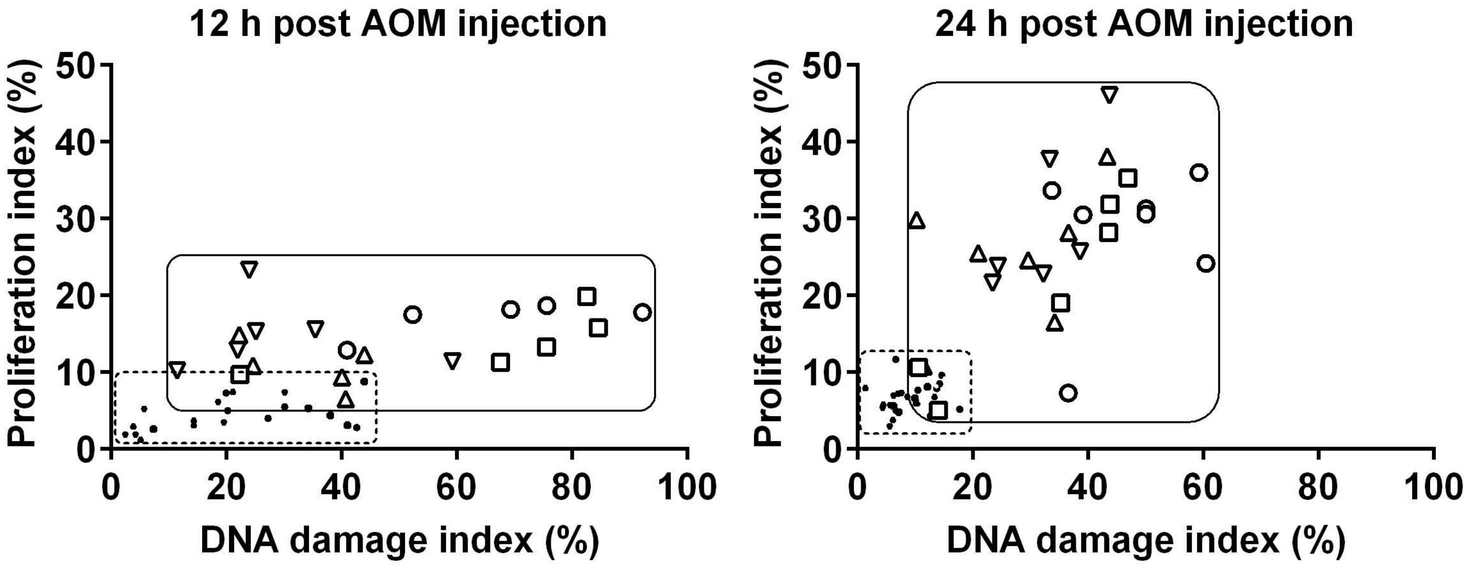

C

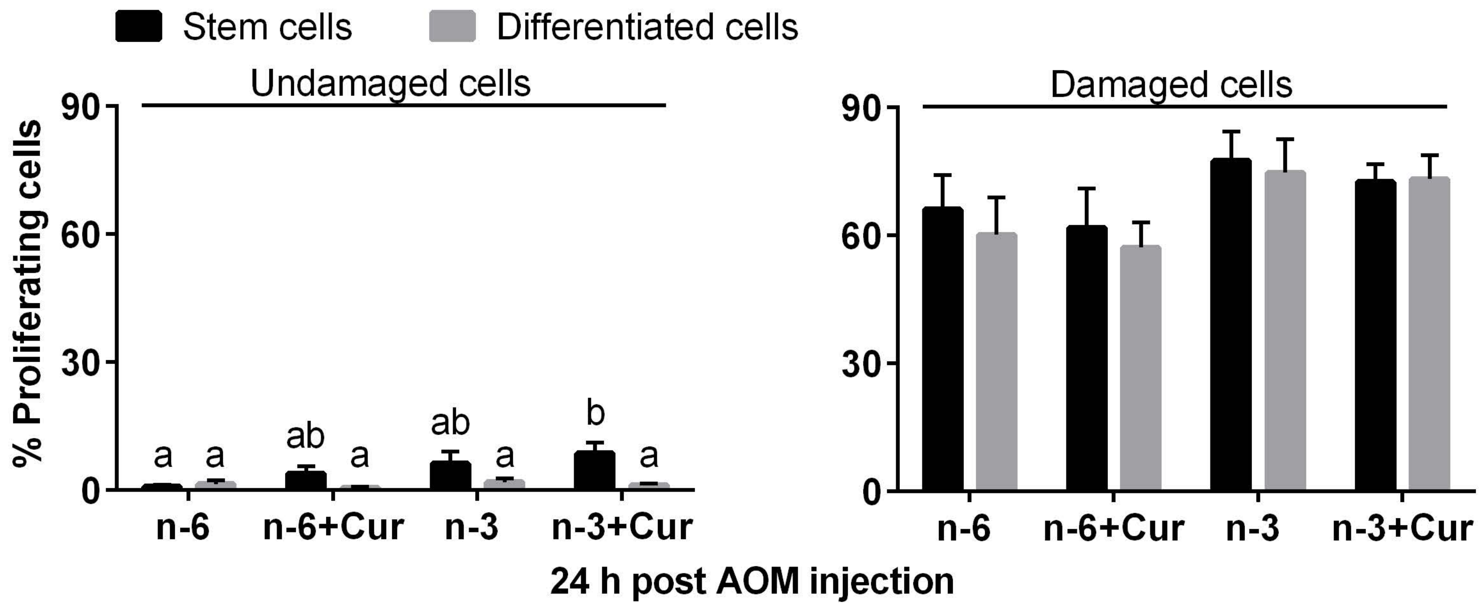

Supplemental Figure 2

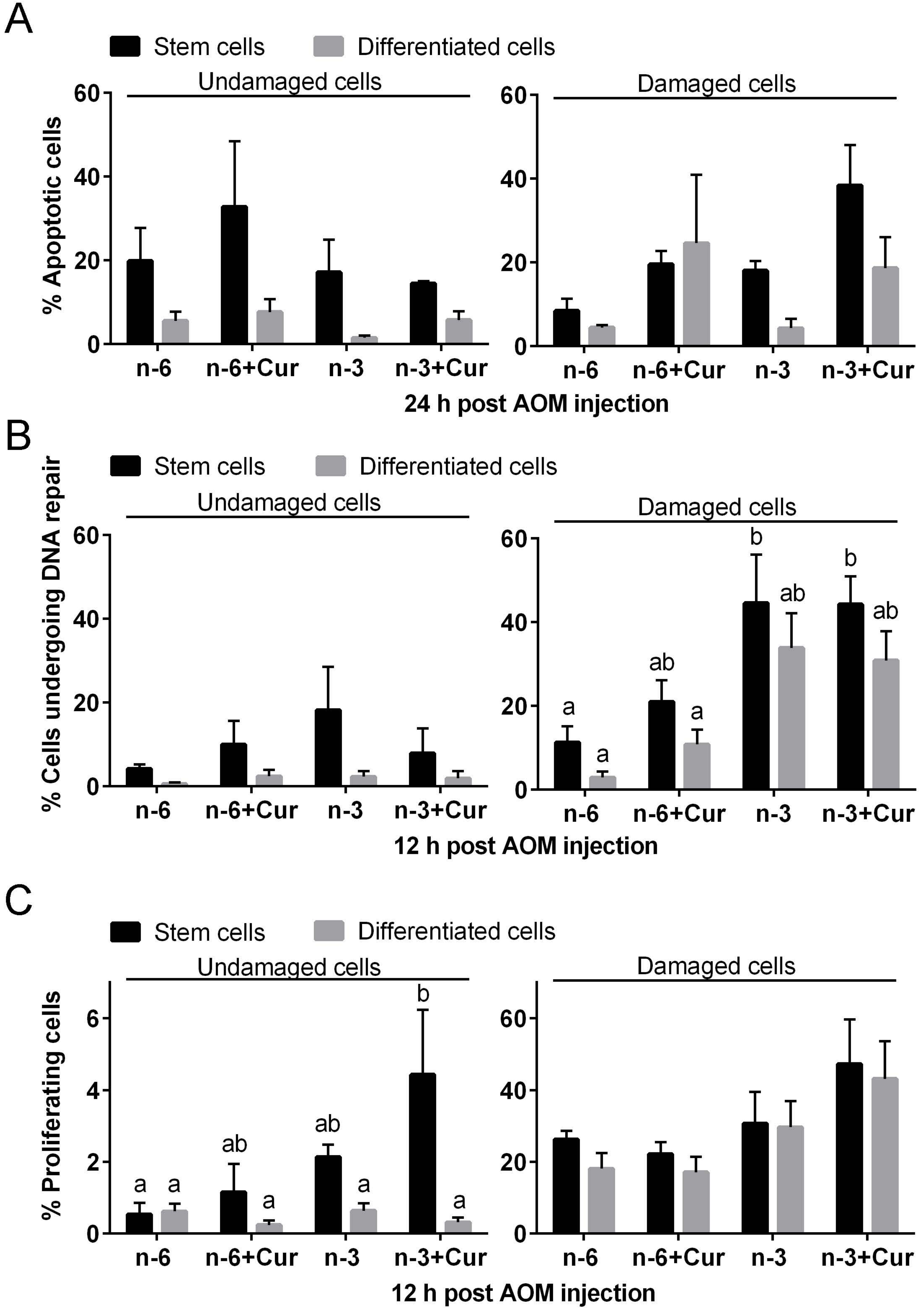

Supplemental Figure 3

A

Comparisons (TukeyHSD test)	Mean difference	Significance (p-value)	95% family-wise confidence level	
			Lower bound	Upper bound
n-3 PUFA vs n-6 PUFA	0.2271	0.062	-0.0088	0.4629
n-6 PUFA+Cur vs n-6 PUFA	0.0682	0.850	-0.1610	0.2974
n-6 PUFA+Cur vs n-3 PUFA	-0.1589	0.256	-0.3881	0.0703
n-3 PUFA+Cur vs n-6 PUFA	0.5775	<0.001	0.3483	0.8067
n-3 PUFA+Cur vs n-3 PUFA	0.3504	0.001	0.1212	0.5796
n-3 PUFA+Cur vs n-6 PUFA+Cur	0.5093	<0.001	0.2869	0.7317

B

Two-way ANOVA - Lgr5⁺ Stem cells					
Source of Variation	Degree of freedom	Sum of squares	Mean squares	F-value	Pr > F
n-3 PUFA	1	1.2048	1.2048	40.032	$5.61x10^{-7}$
Curcumin	1	0.3711	0.3711	12.331	0.00143
n-3 PUFA+Curcumin	1	0.1687	0.1687	5.605	0.02456

Two-way ANOVA - Differentiated cells					
Source of Variation	Degree of freedom	Sum of squares	Mean squares	F-value	Pr > F
n-3 PUFA	1	0.1024	0.1024	3.394	0.0761
Curcumin	1	0.1628	0.1628	5.396	0.0277
n-3 PUFA+Curcumin	1	0.0125	0.0125	0.413	0.5255

C

Comparisons (TukeyHSD test)	Mean difference	Significance	95% family-wise confidence level	
			Lower bound	Upper bound
n-3 PUFA vs n-6 PUFA	-0.0035	0.999	-0.0706	0.0636
n-6 PUFA+Cur vs n-6 PUFA	0.0105	0.961	-0.0522	0.0732
n-6 PUFA+Cur vs n-3 PUFA	-0.0140	0.940	-0.0858	0.0578
n-3 PUFA+Cur vs n-6 PUFA	-0.6754	<0.001	-0.7612	-0.5896
n-3 PUFA+Cur vs n-3 PUFA	-0.6719	<0.001	-0.7646	-0.5792
n-3 PUFA+Cur vs n-6 PUFA+Cur	-0.6859	<0.001	-0.7754	-0.5964

Two-way ANOVA - β-catenin in ACF				
Source of Variation	Estimate	Stand.Error	T-value	Pr > F
n-3 PUFA	-0.0035	0.0231	-0.152	0.881
Curcumin	0.0105	0.0216	0.487	0.634
n-3 PUFA+Curcumin	-0.6824	0.0385	-17.730	$5.47x10^{-11}$

Supplemental Table 1
