# Supplementary material for: Rapidly cycling Lgr5+ stem cells are exquisitely sensitive to extrinsic dietary factors that modulate colon cancer risk
Source: Cell Death Dis. 2016 Nov 10;7(11):e2460–. doi: 10.1038/cddis.2016.269 (PMC5260883; doi:10.1038/cddis.2016.269)
Supplement: Supplementary Information [file cddis2016269x2.docx]

**Supplemental Figure 1. (A)** Experimental diet composition. Timeline of the treatments at **(B)** pre-tumor stage and **(C)** initiation stage of tumorigenesis. **(D)** Food intake and **(E)** body weight gain. **(F)** Amount of fatty acids and curcumin in colonic crypts (n=4). n-6: n-6 PUFA, n-6+Cur: n-6 PUFA+curcumin, n-3: n-3 PUFA, and n-3+Cur: n-3 PUFA+curcumin.

**Supplemental Figure 2.** **Effect of n-3 PUFA±Cur on AOM-induced proliferation in mouse colonic crypts at 12 and 24 h post AOM injection. (A)** Comparison of EdU^+^ (proliferating) stem and differentiated cells in the distal colon of saline, and 12 and 24 h post AOM injected mice (*left*). GFP^+^ crypts (n=5 per diet for saline and n=5-6 per diet for 12 and 24 h post AOM injection) were counted. Refer to Figure 1A legend for statistical details. Different letters indicate significant (p<0.05) differences between treatment groups. **(B)** Association between the AOM-induced proliferating cells and γH2AX^+^ cells in stem and differentiated cells at 12 h (*left*) and 24 h (*right*). Each point represents an individual animal and diets are labeled with different symbols as indicated in the Table in panel A. Data from stem cells and differentiated cells not significantly different from 0 are within the area of the square solid lines and dotted lines, respectively, instead of showing slope since none of the slopes were different from zero. Proliferation index = # of EdU^+^ stem or differentiated cells / total # of stem or differentiated cells per crypt x 100 at 12 and 24 h post AOM injection; Damage index = # of γH2AX^+^ stem or differentiated cells / total # of stem or differentiated cells per crypt x 100 at 12 and 24 h post AOM injection. Refer to Figure 2B legend for statistical details. **(C)** Percentage of undamaged proliferating cells (# of EdU^+^ and γH2AX^-^ stem or differentiated cells / total # of γH2AX^-^ stem or differentiated cells x100) at 24 h post AOM injection (*left*). Percentage of damaged proliferating cells (# of double positive EdU^+^ and γH2AX^+^ stem or differentiated cells / total # of γH2AX^+^ stem or differentiated cells x100) at 24 h post AOM injection (*right*). Refer to Figure 1A legend for statistical details. Different letters indicate significant (p<0.05) differences between treatment groups. n-6: n-6 PUFA, n-6+Cur: n-6 PUFA+curcumin, n-3: n-3 PUFA, and n-3+Cur: n-3 PUFA+curcumin.

**Supplemental Figure 3.** **(A)** Percentage of non-targeted apoptosis (# of TUNEL^+^ and γH2AX^-^ stem or differentiated cells / total # of γH2AX^-^ stem or differentiated cells x 100) at 24 h post AOM injection (*left*). Percentage of targeted apoptosis (# of double positive TUNEL^+^ and γH2AX^+^ stem or differentiated cells / total # of γH2AX^+^ stem or differentiated cells x 100) at 24 h post AOM injection (*right*). Refer to Figure 1A legend for animal number and statistical details. Different letters indicate significant (p<0.05) differences between treatment groups. **(B)** Percentage of undamaged cells expressing MGMT (# of MGMT^+^ and γH2AX^-^ stem or differentiated cells / total # of γH2AX^-^ stem or differentiated cells x 100) at 12 h post AOM injection (*left*). Percentage of damaged cells expressing MGMT (# of double positive MGMT^+^ and γH2AX^+^ stem or differentiated cells / total # of γH2AX^+^ stem or differentiated cells x 100) at 12 h post AOM injection (*right*). Refer to Figure 1A legend for statistical details and Figure 3A for animal numbers. Different letters indicate significant (p<0.05) differences between treatment groups. **(C)** Percentage of undamaged proliferating cells (# of EdU^+^ and γH2AX^-^ stem or differentiated cells / total # of γH2AX^-^ stem or differentiated cells x 100) at 12 h post AOM injection (*left*). Percentage of damaged proliferating cells (# of double positive EdU^+^ and γH2AX^+^ stem or differentiated cells / total # of γH2AX^+^ stem or differentiated cells x 100) at 12 h post AOM injection (*right*). GFP^+^ crypts (n=5 per diet for saline and n=5-6 per diet for 12 and 24 h post AOM injection) were counted. Refer to Figure 1A legend for statistical details. Different letters indicate significant (p<0.05) differences between treatment groups. n-6: n-6 PUFA, n-6+Cur: n-6 PUFA+curcumin, n-3: n-3 PUFA, and n-3+Cur: n-3 PUFA+curcumin.

**Supplemental Table 1. (A&B)** Assessment of statistical interaction between dietary n-3 PUFA and curcumin with respect to the induction of apoptosis in Lgr5^+^ stem cells by Tukey’s HSD test **(C)** and in stem and differentiated cells by two-way ANOVA.
